# Di(2-ethylhexyl) Phthalate Alters Primordial Germ Cell Distribution and the Reproductive Neuroendocrine Regulatory Axis in Zebrafish Embryos

**DOI:** 10.3390/toxics13121032

**Published:** 2025-11-29

**Authors:** Biljana Tesic, Svetlana Fa Nedeljkovic, Zoran Marinović, Zsolt Csenki-Bakos, Maja Marinović, Edward T. Petri, Kristina Pogrmic-Majkic, Bojana Stanic, Nebojsa Andric

**Affiliations:** 1 Department of Biology and Ecology, Faculty of Sciences, University of Novi Sad, Trg Dositeja Obradovica 2, 21000 Novi Sad, Serbia; biljana.tesic@dbe.uns.ac.rs (B.T.); maja@dbe.uns.ac.rs (M.M.); edward.petri@dbe.uns.ac.rs (E.T.P.); kristina.pogrmic@dbe.uns.ac.rs (K.P.-M.); bojana.stanic@dbe.uns.ac.rs (B.S.); nebojsa.andric@dbe.uns.ac.rs (N.A.); 2 Institute of Aquaculture and Environmental Safety, Hungarian University of Agriculture and Life Sciences, Páter Károly u. 1, H-2100 Gödöllő, Hungary; marinovic.zoran@uni-mate.hu (Z.M.); csenki-bakos.zsolt.imre@uni-mate.hu (Z.C.-B.)

**Keywords:** DEHP, PGC, neuroendocrine regulation, reproduction, development, zebrafish (*Danio rerio*), molecular docking, estrogen receptors

## Abstract

Di(2-ethylhexyl) phthalate (DEHP) is known to adversely affect reproduction. Our previous study demonstrated that DEHP exposure during embryogenesis impaired fertility in adult female zebrafish. The objective of this study was to investigate developmental events underlying this effect. Embryos were exposed to DEHP 5 h post-fertilization (hpf), and the distribution of primordial germ cells (PGCs), along with the expression of genes involved in PGC migration, maintenance, and neuroendocrine regulation, was assessed. Molecular docking simulations were performed to evaluate whether DEHP’s main metabolite, mono(2-ethylhexyl) phthalate (MEHP), is able to bind to zebrafish estrogen receptors (Esr). Our results show that DEHP reduced the expression of *cxcr4b*, *cxcr7b*, *esr1*, and *esr2a* at 24 hpf. Using *vasa:egfp* transgenic embryos, we found that DEHP altered the distribution of PGCs. In addition, DEHP inhibited the expression of PGC-specific *dazl*. DEHP also induced the expression of *lhb* and *cyp19a1* and reduced the expression of *esr2a* in 120 hpf larvae, consistent with disruption of the neuroendocrine reproductive axis. Molecular docking indicates that MEHP can bind to the ligand-binding domains of Esr1, Esr2a, and Esr2b. Collectively, the results show that DEHP disrupts both PGC distribution and early neuroendocrine signaling pathways, providing mechanistic insight into reduced fertility in adult female zebrafish following embryonic DEHP exposure.

## 1. Introduction

Di(2-ethylhexyl)phthalate (DEHP) is a commonly used plasticizer found in products made from polyvinyl chloride (PVC) plastics, such as flooring tiles, wall coverings, shower curtains, furniture upholstery, toys and baby products, and medical equipment, such as tubing, syringes, and blood storage bags. It is also used as a plasticizer in other polymers, including rubber and styrene, and as a non-plasticizer in cosmetic products, paints, and lubricating oils (https://www.atsdr.cdc.gov/toxprofiles/tp9.pdf, accessed on 24 September 2025). The toxicity of DEHP has been demonstrated in numerous studies. DEHP is classified as an endocrine disruptor and has been associated with various adverse health effects. Human epidemiological and animal studies show that, depending on the dose and duration of exposure, DEHP can disrupt the function of various organs and even cause lasting damage to the immune system [1], kidneys [2], liver [3], cardiovascular system [4], and thyroid system [5]. Numerous studies have shown that the reproductive system is one of the systems most affected by DEHP exposure [6,7,8,9,10]. Neurobehavioral and reproductive system development can be disrupted even at lower doses of DEHP exposure [11,12,13,14,15]. In living organisms, after entering the body, DEHP is quickly hydrolyzed by carboxylesterases (CE) to the primary metabolite mono(2-ethylhexyl) phthalate (MEHP), which is distributed throughout the body and further metabolized into secondary metabolites [16,17]. This process is highly conserved in vertebrates [18]. MEHP is a bioactive metabolite with various known toxic effects, including endocrine disruption [19,20,21]. Therefore, beginning in the 2000s, regulations on DEHP usage during manufacturing were implemented in most countries worldwide. Limitations on DEHP usage have been set for food containers, medical devices, and especially for children’s articles and toys: the concentration of DEHP cannot exceed >0.1% by weight, and in some regions, like the EU, DEHP is totally prohibited [22].

As postulated by the developmental origins of health and disease concept, unfavorable conditions, including chemical exposure early in life, has the potential to reprogram the development of various organs and can cause lasting altered functionality that can lead to diseases later in life [23]. The reproductive system is one of the first to be affected by chemical exposure because many chemicals, even at low concentrations, have the potential to disturb hormones which control its development [24]. Based on animal studies using rats and mice, as well as aquatic organisms such as zebrafish, developmental exposure to DEHP has been demonstrated to disturb reproduction in adults [7,13,15,25,26,27].

The reproductive system of zebrafish shares significant similarities with that of higher vertebrates. Both the ovaries and testes perform essential functions, including the production of steroid hormones and germ cells such as oocytes and sperm, respectively. This system is regulated by gonadotropins, which are secreted by the pituitary gland and gonadotropin-releasing hormone from the hypothalamus. As in higher vertebrates, the development of zebrafish gonads begins with the specification of primordial germ cells (PGCs), the precursors of germ cells, which occur early during embryogenesis, ~3 h post-fertilization (hpf). Specification and initial maintenance of PGCs are mediated by maternal proteins derived from germplasm, which is inherited from the oocyte. Once PGCs are specified, they migrate to regions where gonads develop [28]. Migration begins during gastrulation, at ~5 hpf, when PGCs employing a bleb-driven amoeboid migration mode are driven by chemotactic cues [29]. Stroma-derived factor 1a (Sdf-1a), which is expressed in embryonic tissue along the pathway, is coupled with c-x-c chemokine receptor type 4b (Cxcr4b), which is expressed on PGCs and enables chemotactic movement [30]. Another PGC receptor, Cxcr7b, also binds Sdf-1a but is expressed in the trailing region of the migrating cells, where it removes Sdf-1a from the tissue and prevents backward PGC movement [31]. Proteins from germplasm, such as Nanos1, are also important for PGC migration [32]. It has been shown that estrogens, via nuclear estrogen receptors 1 (Esr1) and 2a (Esr2a), play an important role in regulating the proper distribution of PGCs during migration [33]. PGCs settle in the gonadal region at 24 hpf. The number of PGCs per individual embryo varies, and it has been shown that embryos with a lower number of PGCs tend to develop as males, and those with a higher number of PGCs develop as females [34]. Numerous genes, such as *vasa*, *nanos1*, *nanos3*, *piwil1*, *piwil2*, *dazl*, *dnd*, and others, are involved in maintaining the function of PGCs, and their transcripts are initially supplied by germplasm. After the embryonic genome is activated at ~3 hpf, zygotic transcription begins for those genes, although at a different rate for each individual gene [35,36,37,38,39]. The PGC number remains constant until 7–10 days post-fertilization (dpf), when they start to proliferate and the development of gonads continues [40].

In parallel with the establishment of PGCs in the gonadal region, neuroendocrine regulation of reproduction is also established. In zebrafish, the gonadotropin-releasing hormone isoform 3 (Gnrh3) is considered to be the primary regulator of pituitary secretion of follicle-stimulating hormone (Fsh) and luteinizing hormone (Lh), similar to the regulatory mechanisms observed in other vertebrates. However, recent findings suggest that additional neuropeptides may also be involved in the regulation of Fsh and Lh secretion, indicating more complex control of reproductive endocrine signaling [41,42]. Expression of *gnrh3* in the zebrafish brain has been detected as early as 25 hpf [43], while adenohypophysis expression of the β-subunit of Fsh (*fshb*) and Lh *(lhb*) was detected at 78 hpf [44]. It is believed that early expression of gonadotropins has a role in general development, especially in the development of the nervous system [45]. Sex hormone receptors, especially estrogen receptors, are an important part of the neuroendocrine regulatory system and are expressed and fully functional during embryonic development. They are responsible for guiding different developmental processes beyond the development of the reproductive system [46,47].

Our previous study showed that exposure to DEHP from 5 dpf to 120 hpf disrupted reproduction in adult female zebrafish by reducing fertility, lowering the gonadosomatic index and blood estradiol levels, and inhibiting oogenesis and the gene expression of estrogen and gonadotropin receptors in the ovaries of adult females [13]. In this study, we aimed to discover the reason behind such effects in adults. Using the same experimental setup, zebrafish embryos were exposed to DEHP from 5 hpf to 120 hpf. The effects of DEHP exposure on PGC distribution and gene expression were investigated, including the expression of genes guiding PGC migration and the expression of genes affecting PGC maintenance and establishment of neuroendocrine regulation of reproduction. Expression of *gnrh3*, *fshb*, *lhb*, steroid hormone receptors, and the critical enzyme aromatase (*cyp19a1*) was monitored during this developmental window.

## 2. Materials and Methods

### 2.1. Zebrafish Husbandry and Breeding

The zebrafish wild-type AB strain was housed in flow-through tanks under standard conditions: temperature 28 ± 1 °C, pH 7.4–7.9, and a photoperiod of 14 h light/10 h darkness at the Department of Biology and Ecology, Faculty of Sciences, University of Novi Sad (Novi Sad, Serbia). The adult fish were fed flake food (Sera Vipan Nature Tropical, Heinsberg, Germany) twice a day and fed live brine shrimp, *Artemia nauplii*, once a day. A group breeding regimen was employed (5 females/3 males, randomly selected) to obtain embryos for the experiment. Collected embryos were rinsed and maintained in ISO water (2 mM CaCl_2_ × 2H_2_O, 0.53 mM MgSO_4_ × 7H_2_O, 0.75 mM NaHCO_3_, and 0.075 mM KCl) before and during exposure. Water was prepared according to ISO standard for freshwater fish water quality for substance toxicity testing, ISO 7346-3:1996, (https://www.iso.org/standard/14030.html, accessed on 24 September 2025). Three hours post-fertilization (hpf), the embryos were sorted under a stereomicroscope (SMZ800N NIKON, Tokyo, Japan). Unfertilized eggs and dead embryos were removed, and only viable embryos were selected for the experiments.

For the purpose of visualizing PGC migration, we used *vasa:egfp* transgenic zebrafish (*ddx4^sa6158/sa6158^*). The *vasa* (*ddx4*) gene encodes a DEAD-box RNA helicase that is specifically expressed in germline cells and plays a critical role in germ cell development and maintenance, thus making it a suitable model for visualization of primordial germ cell (PGC) migration in vivo. *Vasa:egfp* fish were housed in a recirculated zebrafish housing system (Tecniplast Zebtec, Techniplast, Buguggiate, Italy) at the Institute for Aquaculture and Environmental Safety, Hungarian University of Agriculture and Life Sciences (Gödöllő, Hungary). Adult fish were maintained at 25 ± 0.5 °C, pH 7.0 ± 0.2, conductivity 525 ± 50 μS (system water), and 14 h light/10 h dark cycle. Fish were fed SDS Small Gran granulated feed twice a day, with an additional feeding of *Artemia nauplii* once a day. To produce larvae, adult fish were placed in breeding tanks in the late afternoon the day before spawning. The subsequent morning, dividing walls were removed, and fish were allowed to spawn naturally. Embryos were then collected, placed in a Petri dish (10 cm in diameter) filled with E3 medium, and grown in an incubator at 25 ± 0.5 °C. Unfertilized eggs and dead embryos were removed, and only viable embryos between the 4 and 16 cell stages were selected under a dissecting light stereomicroscope (Leica Microsystems GmbH; Wetzlar, Germany) for subsequent toxicological assays.

### 2.2. Zebrafish Embryo Exposure

Viable embryos were randomly distributed in Ø55 Petri dishes, with 20 embryos per dish. DEHP stock solutions (analytical standard, Sigma Aldrich, Steinheim, Germany) were prepared in dimethyl sulfoxide (DMSO, Sigma Aldrich, Steinheim, Germany). Environmentally relevant concentrations of DEHP solutions were used for exposure: concentrations of 10, 100, and 1000 nmol DEHP/L were prepared in ISO water or E3 medium (depending on the fish strain used, for different purposes) by diluting the stock solutions. The percentage of DMSO in all groups, including the solvent control group, was 0.1%. Each exposure Petri dish contained 10 mL of either the solvent control or DEHP solution. Exposure was initiated at 5 hpf and lasted until 24 hpf or 120 hpf (5 days post-fertilization, 5 dpf) at 27 °C for the AB strain and at 25 °C ± 0.5 °C for the *vasa:egfp* line, with a photoperiod of 14 h light/10 h darkness. One-half of the control and each DEHP solution were renewed daily. If coagulated embryos were noted, they were excluded from further treatment. After exposure ended at 24 hpf and 120 hpf, the embryos and larvae of the AB strain were washed with ISO water, pipetted into 1.5 mL tubes with 5 embryos or larvae per tube, and euthanized by removing water and placing the tubes on dry ice for several minutes. Frozen embryos and larvae of the AB strain were stored at −80 °C for RNA extraction, while transgenic larvae used for PGC visualization were washed with E3 medium and immediately observed and photographed under a fluorescent stereomicroscope.

### 2.3. Imaging and Analyzing PGCs

In order to count PGCs and analyze the migration process, PGCs were visualized using the *vasa:egfp* transgenic line at the 120 hpf larval stage under a Leica M205 FA stereomicroscope (Leica DFC 7000T camera, Leica Application Suite X, Leica Microsystems GmbH; Wetzlar, Germany). Larvae were anesthetized in a 0.02% MS-222 solution and subsequently transferred into a Petri dish filled with 5% methyl cellulose to enable precise positioning. They were placed on their sides, pushed to the bottom of the methyl-cellulose gel, and positioned using a micro-loader pipette tip (Eppendorf, Hamburg, Germany). They were placed first on their right side and later on their left side in order to more accurately visualize the entire PGC. Images were recorded at a magnification of 16×, with an exposure time under bright field set at 40 ms, and the exposure time under the fluorescent GFP filter was set at 1.5 s. In order to count the total number of PGCs, images of both sides of the body were analyzed regardless of their position or distance from the region of the future gonad. PGCs which were not positioned at the gonadal region were noted. For each individual fish, the percentage of PGCs outside of the gonadal region was calculated. Finally, average percentages of PGCs outside of the gonadal region were calculated for each group. A total of 10 larvae per group per experiment were observed and photographed. The experiment was repeated 3 times.

### 2.4. RNA Extraction and RT-qPCR Analysis

Total RNA was extracted from 5 zebrafish embryos (24 hpf) or larvae (120 hpf) per group, per experiment, using NZYol reagent (NZYTech, Lisboa, Portugal) according to the manufacturer’s instructions. The concentration and purity of the extracted RNA was determined using a BioSpec-Nano (Shimadzu Scientific Instruments, Kyoto, Japan). The A260/A280 ratio was in the range of 1.8–2.0. A High-Capacity cDNA Reverse Transcription Kit (Applied Biosystems, Foster City, CA, USA) was used to reverse-transcribe 500 ng of total RNA into cDNA in a 20 μL reaction volume. Quantitative real-time PCR (RT-qPCR) was performed as described in our previous work, Tesic et al. [13], using a Power SYBR Green master mix (Applied Biosystems Foster City, CA, USA). Melting curve analysis was performed to ensure the specificity of the primers. Zebrafish-specific primers were designed for the relevant genes (*elf1a*, *cxcr4b*, *sdf-1a*, *cxcr7b*, *nanos1*,*esr1*, *esr2a*, *esr2b*, *piwil1*, *piwil2*, *dazl*, *lhb*, and *gnrh3*) using the Primer-Blast online primer designing tool from NCBI (https://www.ncbi.nlm.nih.gov/tools/primer-blast/, accessed on 1 September 2020), while the sequences of primers for the *fshb* gene were obtained from a publication [48]. As in our previous study [13], *elf1a* was used as a reference gene. A complete list of primer sequences, their efficiencies, amplicon lengths, and accession numbers is given in Table 1. Relative expression of the investigated genes was calculated using the ΔΔCt method [49]. For gene expression analyses, the experiment was repeated 4–6 times.

### 2.5. Molecular Docking Simulations

Molecular docking simulations were conducted using the Webina server, version 1.0.5., which uses the AutoDock Vina algorithm [50,51]. PDB coordinates for MEHP were extracted from PDB entry 8BF2 [52]. Addition of hydrogens, geometry optimization, and energy minimization were performed in Avogadro, version 1.2.0. [53]. Models of zebrafish estrogen receptors, Esr1, Esr2a, and Esr2b, were made from FASTA sequences (UniProt codes P57717, Q7ZU32, and Q5PR29) using AlphaFill software, version 2.3.0 [54]. Esr1 was aligned with human ERα (PDBID: 1a52) [55], while Esr2a and Esr2b were aligned with human ERβ (PDBID: 3OLS) [56]. Ligand-binding domains were estimated from the H3 position (V/IMxxWAKxxP sequence), which is conserved among ERs, to H12 (at the C-terminus) [57] and extracted to separate PDB files for docking simulations. The position of docking boxes was estimated based on alignment to structures of the human estrogen receptor ligand-binding domain co-crystalized with estradiol (PDBID: 1A52, 3OLS). Box sizes were 20 × 20 × 20 Å, and Webina coordinates for box center were x = 107, y = 15, and z = 97; x = 27, y = −26, and z = −11; and x = 26, y = −28, and z = −12 for Esr1, Esr2a, and Esr2b, respectively. Estradiol was used as a control ligand in all simulations. Exhaustiveness was set to 64 to maximize accuracy. Webina returned estimated binding energies [kCal/mol] and docking models that were inspected visually in PyMol, version 3.1.6.1. (https://legacy.ccp4.ac.uk/newsletters/newsletter40/11_pymol.pdf, accessed on 15 September 2025). Protein-Ligand Interaction Profiler (PLIP) tool [58] was used to detect non-covalent bonds between docked ligands and the receptors.

### 2.6. Statistical Analysis

All results are presented as the mean ± standard error of the mean (SEM). Statistical analysis was performed using a one-way analysis of variance (ANOVA) followed by Dunnett’s post hoc test using the Prism8 software package (GraphPad Prism 8 Software package, Inc., La Jolla, CA, USA). Significant differences were considered to be *p*-values < 0.05.

## 3. Results

### 3.1. DEHP Inhibits Expression of Genes Involved in PGC Migration and Distribution

**Figure 1:**
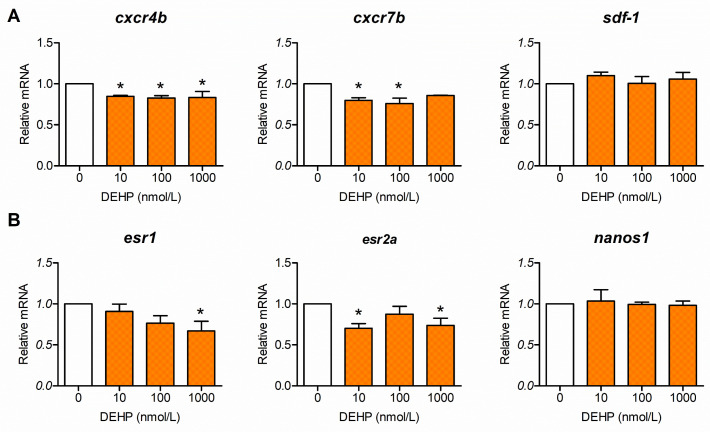
Effect of DEHP on the expression of genes involved in PGC migration. Zebrafish embryos were exposed to different concentrations of DEHP ranging from 5 hpf to 24 hpf. RT-qPCR was used to evaluate relative mRNA for (**A**) genes directly involved in chemotactic movement of PGC and (**B**) genes that affect PGC migration and distribution. Experiments were repeated 5 times. The results are expressed as the mean ± SEM. White bars represent the control, while orange bars represent groups exposed to different DEHP concentrations. * *p*-value < 0.05 vs. control group.

To evaluate the expression of genes involved in the PGC migration process, zebrafish embryos were exposed to DEHP at concentrations of 0, 10, 100, and 1000 nmol/L: beginning at 5 hpf, the onset of PGC migration, until 24 hpf, when this process is completed. Our results revealed that DEHP exposure significantly inhibited the expression of receptors involved in the chemotactic guidance of PGCs, *cxcr4b* and *cxcr7b* (around 15% and 20% of the control value). All DEHP concentrations reduced the expression of *cxcr4b*, whereas the expression of *cxcr7b* was affected at 10 and 100 nmol DEHP/L. The gene expression of stroma-derived factor 1a (*sdf-1a*), which binds to Cxcr4b and Cxcr7b, was unaffected (Figure 1A). The gene expression of estrogen receptors *esr1* and *esr2a*, which are involved in the proper distribution of PGCs, was inhibited. *Esr1* was inhibited at 1000 nmol DEHP/L, while *esr2a* was inhibited at 10 and 1000 nmol DEHP/L. The relative level of *nanos1* transcripts involved in migration was unaffected by DEHP exposure (Figure 1B).

### 3.2. DEHP Alters Distribution of PGCs

**Figure 2:**
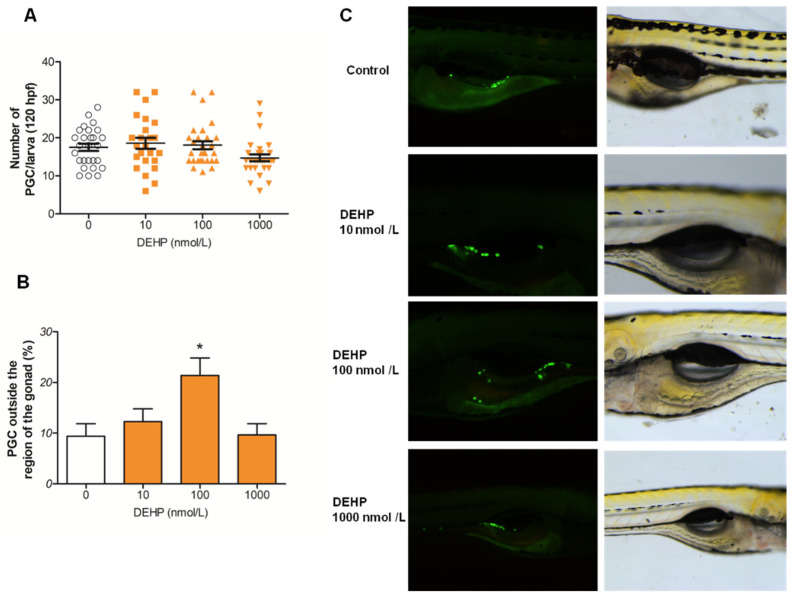
Effect of DEHP on PGC number and distribution. Transgenic *vasa::egfp* zebrafish embryos were exposed to different concentrations of DEHP from 5 hpf to 120 hpf and imaged using fluorescent microscopy. (**A**) Number of visible PGCs. (**B**) Percentage of PGCs outside of the gonadal region. (**C**) Images of PGC distribution in 120 hpf larvae. The experiment was repeated 3 times, and the total number of larvae analyzed per group was 24–29. The results are expressed as the mean ± SEM. White bar and dots represent control, while orange bars and dots represent groups exposed to different DEHP concentrations. * *p*-value < 0.05 vs. control group.

To assess the number and distribution of PGCs under DEHP exposure, *vasa:egfp* transgenic zebrafish embryos were exposed to DEHP from 5 hpf to 120 hpf. The effects of DEHP were evaluated at the larval stage (120 hpf). Our results demonstrate that DEHP did not alter the total number of PGCs observed in larvae (Figure 2A). However, DEHP exposure disrupted their proper localization, with a significantly higher percentage of PGCs positioned outside of the gonadal region in the 100 nmol/L DEHP treatment group compared to the control (Figure 2B,C).

### 3.3. DEHP Affects Gene Expression Related to Maintenance and Functionality of PGCs

**Figure 3:**
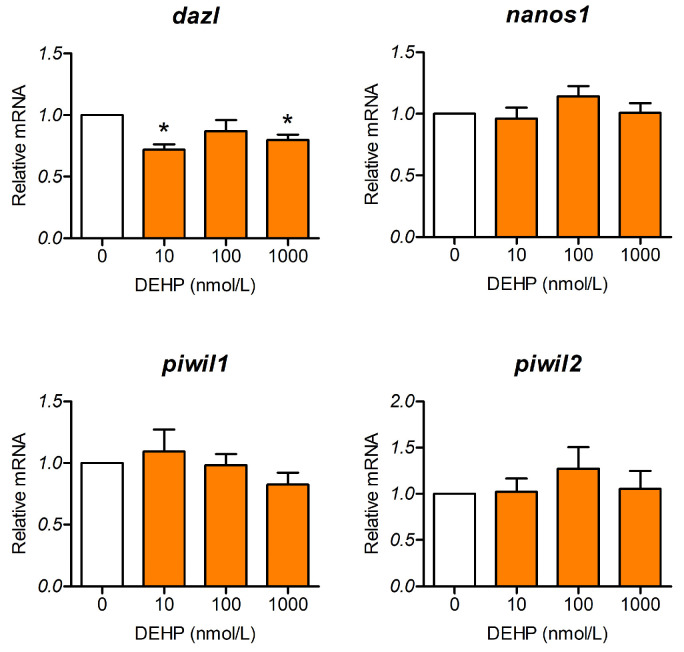
Effect of DEHP on expression of genes affecting PGC maintenance and function. Zebrafish embryos were exposed to different concentrations of DEHP between 5 hpf and 120 hpf. RT-qPCR was used to evaluate relative mRNA. The experiment was repeated 6 times. The results are expressed as the mean ± SEM. White bars represent control, while orange bars represent groups exposed to different DEHP concentrations. * *p*-value < 0.05 vs. control group.

To validate whether DEHP affects the maintenance and functionality of PGCs, we evaluated transcripts of specific crucial genes, such as *piwil1*, *piwil2*, *nanos1*, and *dazl*, in 120 hpf larvae. Our results show that DEHP significantly decreased the relative amount of *dazl* transcripts in the 10 and 1000 nmol DEHP/L groups, whereas the remainder of the transcripts were unaffected by DEHP exposure (Figure 3).

### 3.4. DEHP Alters Expression of Genes Comprising the Reproductive Neuroendocrine Regulatory Axis

**Figure 4:**
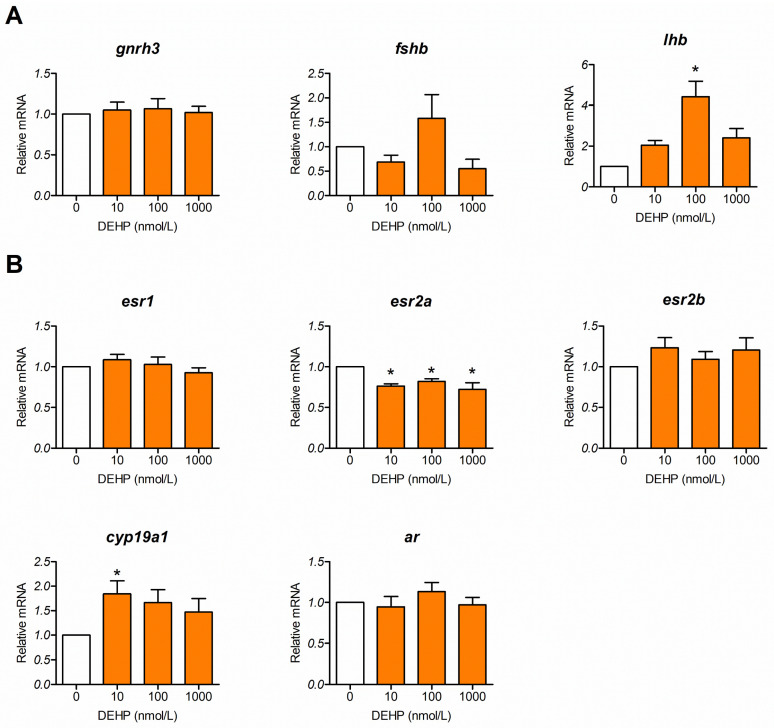
Effect of DEHP on the expression of genes involved in neuroendocrine regulation of reproduction. Zebrafish embryos were exposed to different concentrations of DEHP from 5 hpf to 120 hpf. RT-qPCR was used to evaluate relative mRNA for (**A**) hypothalamic and pituitary hormones and their β-subunits and (**B**) genes encoding sex hormone receptors and aromatase. The experiment was repeated 4–6 times. The results are expressed as the mean ± SEM. White bars represent control, while orange bars represent groups exposed to different DEHP concentrations. * *p*-value < 0.05 vs. control group.

Next, we analyzed effects of different concentrations of DEHP on the expression of genes important for the neuroendocrine regulation of reproduction in 120 hpf larvae. We analyzed gonadotropin releasing hormone 3 (*gnrh3*), the β-subunit of follicle-stimulating hormone (*fshb*), the β-subunit of luteinizing hormone (*lhb*), estrogen receptors (*esr1*, *esr2a*, and *esr2b*), aromatase (*cyp19a1*), and the androgen receptor (*ar*). Our results show that the expression of *gnrh3* remains unaffected by DEHP. Expression of *lhb* in the 100 nmol DEHP/L group was significantly induced. The expression of *fshb* showed a slight decrease at 10 and 1000 nmol/L DEHP and an increase at 100 nmol/L DEHP; however, none of these changes reached statistical significance (Figure 4A). On the other hand, expression of *esr2a* was significantly inhibited in all groups exposed to DEHP, while the expression of *esr1* and *esr2b* remained unchanged. The expression of *cyp19a1* was significantly induced by 10 nmol/L DEHP, whereas the expression of *ar* was unaffected (Figure 4B).

### 3.5. Molecular Docking of MEHP to Models of Esr1, Esr2a, and Esr2b

**Figure 5:**
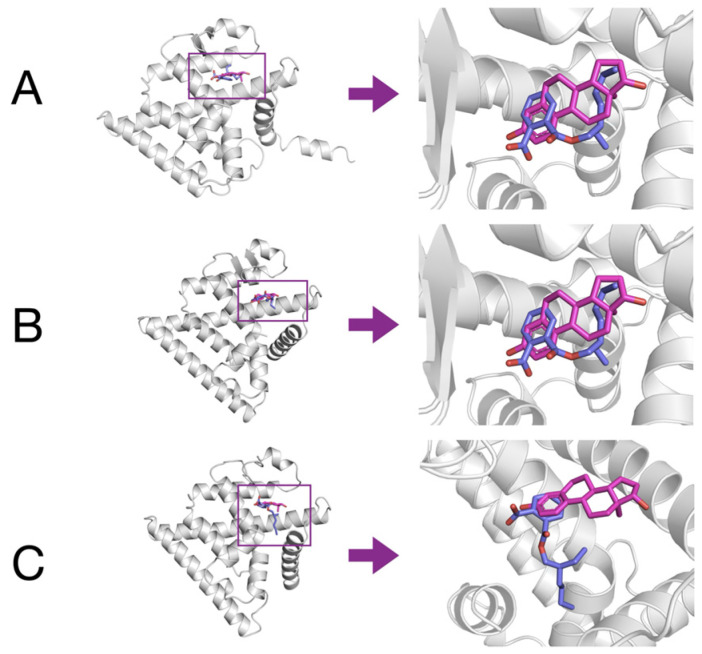
Modeled ligand-binding domains of zebrafish Esr1 (**A**), Esr2a, (**B**) and Esr2b (**C**) with docked estradiol (magenta sticks) and MEHP (blue sticks) overlay. Receptors are represented by gray cartoon.

**Figure 6:**
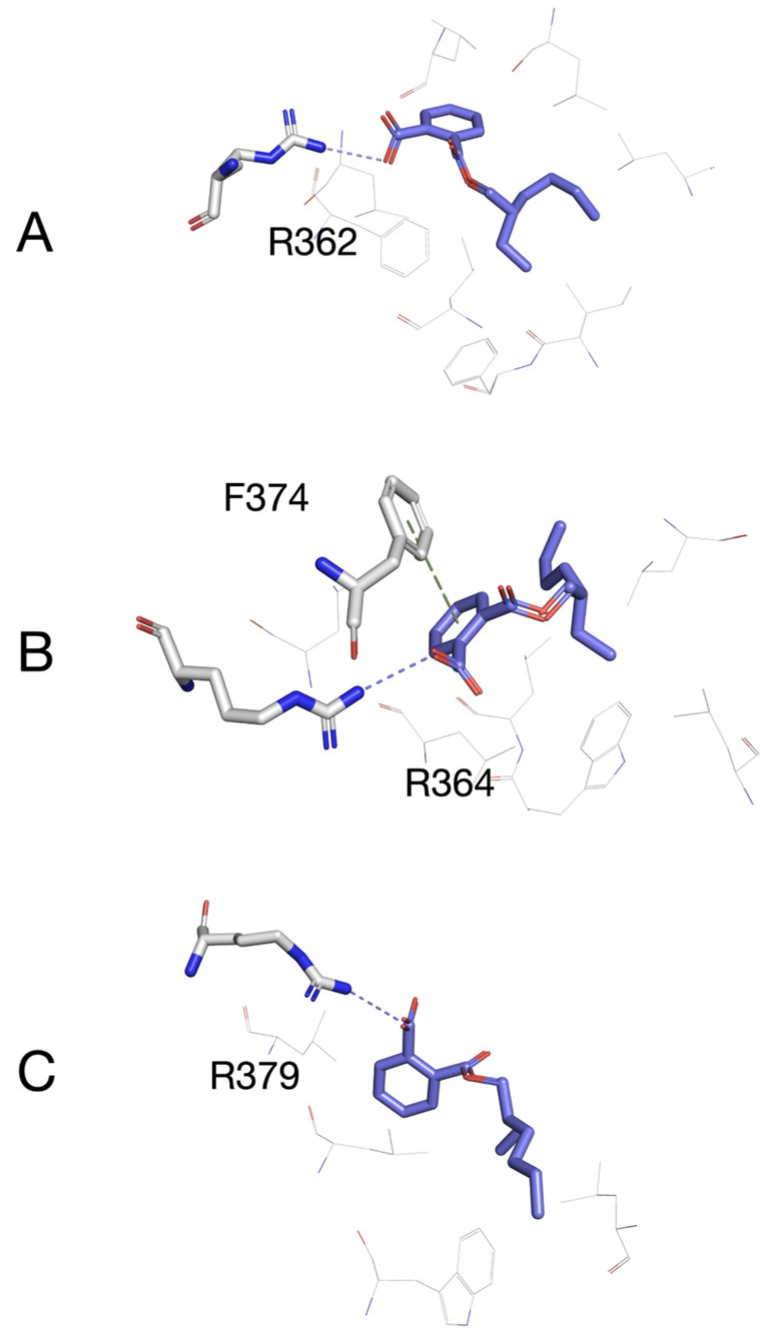
Molecular docking of MEHP (blue sticks) to modeled ligand-binding domains of zebrafish Esr1 (**A**), Esr2a, (**B**) and Esr2b (**C**). Residues that are involved in polar (blue dashes) or stacking (green dashes) interactions with the ligand are labeled and depicted as gray sticks. Residues that form hydrophobic interactions with the ligand are presented as gray lines.

Molecular docking simulations were performed using MEHP, the main DEHP metabolite, since enzymes involved in this transformation are already active in zebrafish embryos [18,59]. Control docking simulations were performed using estradiol as a control ligand for the modeled Esr1, Esr2a, and Esr2b. Results were compared to the positions of estradiol in the structures of the human ligand binding domains of ERα (PDB: 1A52) and ERβ (PDB: 3OLS). It was confirmed for all of the zebrafish isoforms that estradiol was positioned in the same place and orientation as seen in human receptors. Estimated binding energies after docking of estradiol and MEHP to modeled ligand-binding domains of zebrafish estrogen receptors Esr1, Esr2a, and Esr2b are shown in Table 2. Webina suggested that MEHP binds all zebrafish nuclear estrogen receptors with 20–30% weaker affinity than estradiol. Docking models of MEHP to Esr1, Esr2a, and Esr2b are shown overlaid with estradiol in Figure 5, while Figure 6 shows a possible binding mechanism of MEHP with Esr1, Esr2a, and Esr2b. We can see conserved arginine residues (R362, R364, and R379 for Esr1, Esr2a, and Esr2b, respectively), together with a network of hydrophobic bonds, which contribute to binding MEHP to the ligand-binding sites of models of the zebrafish estrogen receptors. In Esr2a, a possible pi-pi stacking interaction between F374 and the phthalate ring of MEHP was observed.

## 4. Discussion

This study builds upon our previous research, which demonstrated that embryonic exposure to DEHP (from 5 to 120 hpf) disrupts reproductive function in adult female zebrafish [13]. In the present work, we aimed to uncover the mechanisms governing the reproductive toxicity of DEHP exposure in adult female zebrafish. We investigated the effects of DEHP on the migration of PGCs, which colonize the gonadal region during this developmental window, as well as on the establishment of neuroendocrine regulation of reproduction, which begins during early embryogenesis.

Zebrafish embryos were exposed to DEHP, beginning at 5 hpf, coinciding with the initiation of PGC migration and continuing until 120 hpf. To investigate early molecular changes, gene expression was analyzed at 24 hpf. The results show that DEHP exposure is associated with significant downregulation of the expression of *cxcr4b* and *cxcr7b*, which encode chemokine receptors expressed on the surface of PGCs. In contrast, the gene expression of their corresponding ligand, Sdf-1a (also known as Cxcl12a), which is produced by somatic cells along the migratory path, remained unaffected. This reduction in *cxcr4b* and *cxcr7b*, despite normal levels of *sdf-1a* transcripts, could negatively affect PGC migration. It has been shown that in embryos in which Cxcr7 function is compromised, PGCs exhibit strongly impaired cell polarity and faulty migration [60]. Cxcr7 acts primarily in the somatic environment rather than directly on PGCs by controlling the shape of the Sdf-1 tissue gradient [61]. Therefore, the lower level of *cxcr7b* expression in this study could alter the Sdf-1a gradient, which would in turn interfere with proper directed migration despite the normal RNA expression pattern of *sdf-1a*. In addition to this, lower levels of *cxcr4b* expression may also contribute to the abnormal migration of PGCs observed in this study since PGCs that do not receive an Sdf-1a signal exhibit a lack of directional movement toward their target [30].

We also showed that DEHP significantly inhibits expression of estrogen receptors in 24 hpf zebrafish embryos. These results are quite interesting since it was demonstrated in zebrafish that the activity of Esr1, but not Esr2, is required for normal PGC migration and distribution. However, the adverse effects of high concentrations of environmental estrogen, 17α-ethinylestradiol, on PGC distribution, were shown to be mediated through Esr2a [33]. The relationship between estrogen receptors and chemokine receptors involved in the cell migration process was demonstrated using migrating zebrafish primordium, where inactivation and overexpression of *esr1* were employed. The study showed that Esr1 acts as a repressor of *cxcr4b* gene expression. The role of Esr1 is to restrict expression of *cxcr4b* to the leading region of the migrating cells and inhibit it in the trailing region, which in turn enables *cxcr7b* expression to occur in this region. In the absence of Esr1, *cxcr7b* is not expressed and PGCs might also move backward instead of only forward, while overexpression of *esr1* would reduce *cxcr4b* expression not only in the trailing region but also in the leading region and inhibit the process of forward migration, which, in both cases, causes aberrant cell migration [62]. Chemicals like bisphenol A, which is well-known for its estrogenic activity, were also shown to cause aberrant distribution of PGCs in zebrafish [63]. The same effects on PGC distribution were detected when zebrafish embryos were exposed to the pesticide endosulfan and the surfactant nonylphenol [33,64]. In our experiments using *vasa:egfp* transgenic embryos, altered distribution of PGCs was further confirmed in the group treated with 100 nmol/L DEHP. It should be noted that the DEHP concentration associated with abnormal PGC localization significantly altered the expression of *cxcr4b and cxcr7b* but did not affect the expression of *esr1* or *esr2a*. However, DEHP concentrations that did not affect PGC distribution (10 and 1000 nmol/L) inhibited the expression of *cxcr4b*, *cxcr7b*, and *esr2a*. Our findings suggest that reduced *esr2a* expression may alleviate the impact of decreased *cxcr4b* and *cxcr7b* levels on the aberrant PGC distribution observed in DEHP-exposed zebrafish, highlighting a potentially protective role of *esr2a* in preserving normal germ cell migration. This is in accordance with the earlier mentioned findings regarding the role of Esr2a in aberrant PGC migration [33]. The inhibited expression of *cxcr4b* and *cxcr7b* might be the result of direct DEHP/MEHP activation of estrogen receptors (Esr1, Esr2a, and Esr2b) but could also be the result of DEHP directly acting on chemokine receptors. The estrogenic activity of DEHP has previously been demonstrated in a 24 h assay using ChgH-EGFP transgenic medaka (*Oryzias melastigma*) eleutheroembryos [65]. In the work of Hamid et al. [66], using a molecular docking approach, it was shown that DEHP itself is able to bind to Esr1 and Esr2. Since DEHP is rapidly metabolized to MEHP by the catalytic activity of carboxyl esterase and lipase, and the toxicity of DEHP is considered to be mediated by MEHP rather than the parent compound [67,68,69], we decided to analyze the interaction of MEHP with the estrogen receptor. We recognize that embryonic metabolism could be limited and that there is no direct evidence of embryonic transformation of DEHP to MEHP. Yet, zebrafish embryos are maternally supplied with carboxylesterase lipase involved in utilization of yolk lipids [70]. The activity of carboxylesterases (CE) involved in xenobiotic transformation is already detectable from the 12 hpf stage [59]. It was also suggested that zebrafish embryonic CE activity could be a suitable biomarker of xenobiotic exposure [59]. In 120 hpf zebrafish larvae, CE activity is observed in the liver, gut and ear [71]. Therefore, it is reasonable to expect that at least a portion of DEHP is transformed into MEHP in zebrafish embryos and larvae. Our molecular docking analyses revealed that MEHP could also bind to zebrafish Esr1, Esr2a and Esr2b, further suggesting that activation of the estrogen receptor can take place in DEHP-exposed zebrafish embryos. Our results indicate that MEHP binds to estrogen receptors with lower affinity compared to E2, while in the work of Hamid et al., it was shown that DEHP binds Esr1 and Esr2 with higher affinity than E2. If we examine the docking positions and contacts formed between MEHP and the estrogen receptors (Figure 6), a conserved hydrogen bond between the guanidinium group of arginine and the carbonyl oxygen of MEHP is visible. These arginine residues are R362 for Esr1, R364 for Esr2a, and R379 for Esr2b. These arginine residues correspond to R394 in ERα and R346 of ERβ and are all involved in binding of a hydroxyl group on the C3 position of the A ring of the estradiol molecule [55,57]. Binding to specific arginine residues was observed in molecular docking studies of phthalate esters, including DEHP binding to models of zebrafish estrogen receptors, as observed by Hamid et al. [66]. Because biotransformation of DEHP to MEHP in early zebrafish embryos is insufficiently characterized, our results should be interpreted with caution regarding MEHP-mediated mechanisms. In general, it is possible that alteration in the expression of chemokine and estrogen receptors and estrogen receptor activation cause misplacement of PGCs outside of the gonadal region, leading to loss of their identity and thus becoming apoptotic or transdifferentiated into other tissues [72]; thus, the region of the future gonad has less PGCs. The aberrant migration and lower number of PGCs in gonads might lead to male-biased sex ratios, considering that PGC numbers at the embryonic stage of zebrafish are predictive of future sex and that early depletion of PGCs favors testis formation [34,73]. Only the total depletion of PGCs would have resulted in infertility and an all-male phenotype [74]. Besides altered PGC migration, in 5 dpf zebrafish larvae, we detected inhibition of PGC-specific expression of *dazl*, which is known to regulate cystogenesis, entrance into meiosis, and specification of germline stem cells in the later stages of development [39]. This might also be an important finding if such disrupted expression is preserved later in development, which could result in inhibited oogenesis.

The effect of DEHP on the neuroendocrine regulatory system was investigated in 120 hpf larvae, when hypothalamic and pituitary components are being differentiated and are producing regulatory hormones [44,75]. Our results show that the expression of some reproductive neuroendocrine-related genes, such as *lhb* and *cyp19a1*, were increased, while others were inhibited *(esr2a*), and some, such as *gnrh3*, *fshb*, *esr1*, *esr2b*, and *ar*, were unchanged in the various treatment groups. Similar analysis was performed after zebrafish embryo exposure to BPA, where significant induction of *gnrh3* and *lhb* expression was detected both in 25 hpf and 120 hpf embryos/larvae, while *fshb* was induced only in 120 hpf larvae and only at the highest concentration of BPA [48]. The lack of change in *gnrh3* after DEHP exposure is not unexpected, considering that its role in fish reproduction has been questioned recently, and new players such as satiety peptide hormone cholecystokinin (CCK) have been proposed to play critical roles in the regulation of fish gonadotropin secretion [41,42,76]. In our study, we noticed that the expression of *lhb* was more sensitive to DEHP exposure than the expression of *fshb*, suggesting that the expression of this mRNA might be the main target of neuroendocrine disruption by DEHP. The estrogenic compound BPA primarily targets *lhb* rather than *fshb*, while its analog BPS has more pronounced effects on *fshb* [48]. We can argue that the expression of *fshb* was also increased after exposure to 100 nmol DEHP/L; however, this change did not reach statistical significance. The potential reason for more pronounced *lhb* mRNA changes can be explained by the fact that estrogens stimulate pituitary *lhb* expression, which can be referred to as a positive feedback loop, similar to what was shown in pubertal female zebrafish [77]. Such a mechanism can also be expected at this early stage and can depend on the estrogenic effects of DEHP [78]. Our results also confirm the estrogenic potency of DEHP. We detected that DEHP stimulated the expression of *cyp19a1*, an enzyme critical for estrogen synthesis.

Analysis of three nuclear estrogen receptors and androgen receptors showed that *esr2a* was inhibited by DEHP in 24 hpf and 120 hpf embryos/larvae. This result suggests that DEHP exposure consistently suppresses *esr2a* expression during early zebrafish development. Unlike the effect on *esr2a*, DEHP inhibits *esr1* expression only at 24 hpf. Others have also analyzed the expression of estrogen receptors in zebrafish larvae after DEHP exposure, and depending on the tested concentration range, different results were obtained. Lee et al. [79] showed that a lower concentration of DEHP (25.6 nmol/L) significantly inhibited the expression of *esr1*, while higher concentrations (256 nmol/L and 2560 nmol/L) had no effect on its expression after exposing zebrafish embryo/larvae for 7 days. Hamid et al. [66] found no effect on *esr1* expression when treating with 64 nmol/L DEHP, but they detected stimulation of *esr1* expression with 128 nmol/L DEHP exposure in 7 dpf larvae. Mu et al. [80] used a similar concentration range and detected no effect with 128 nmol/L DEHP and an increase in the expression of *esr1* only with 640 nmol/L DEHP in 96 hpf larvae These data suggest that DEHP-induced stimulation of *esr1* expression is restricted to certain concentration ranges and depends on the experimental setup. On the other hand, in the work of Lee et al. [79], expression of *esr2b* was not affected, while Hamid et al. showed stimulation of *esr2* expression but did not analyze the specific effects on *esr2a* and *esr2b* forms [66]. The results obtained in our study on *esr2a* expression align with the action of estradiol in cultured zebrafish follicular cells, where this steroid upregulates *esr1* while downregulating *esr2a* and *esr2b* expression [81]. Despite some differences compared to other studies, our results confirm that DEHP can interfere with estrogenic signaling during early zebrafish development.

We note that DEHP-induced changes in gene expression and PGC migration followed a nonlinear dose–response. Several mechanisms could underlie such patterns, most commonly the presence of multiple molecular targets with opposing effects on shared pathways, negative feedback regulation, and high-dose receptor desensitization, among others [82]. We also detected nonlinear effects in adult females following embryonic exposure to the same DEHP concentrations in our previously published study [13], which closely parallel the effects observed in this study.

Using the results of this study, we can propose a link between early DEHP exposure in zebrafish embryos and reproductive dysfunction observed in adult females. Early exposure to DEHP led to abnormal distribution of PGCs, which might have contributed to the impaired gonad function observed in adult females [13]. On the other hand, disruptions in the development of the neuroendocrine system that regulate reproduction, particularly stimulation of *lhb* and persistent inhibition of *esr2a*, could have caused inhibited expression of ovarian gonadotropin receptors *fshr* and *lhr* and estrogen receptors *esr1*, *esr2a*, and *esr2b* and contributed to the inhibited oogenesis observed in adult females [13]. However, not all early molecular changes appear to align with adult phenotypes. For instance, expression of *cyp19a1*, which is upregulated by DEHP in larvae, is in contrast with the reduced estradiol levels observed in adults. This can also be explained by the disturbed neuroendocrine regulation and overall inhibition of ovarian functions seen in adult ovaries. We also detected that a concentration of 100 nmol DEHP/L caused the most adverse effects in zebrafish embryos and larvae, similar to what was noted in adult females [13]. Altogether, the findings from this study offer a more comprehensive understanding of the developmental origins of DEHP-induced reproductive toxicity in zebrafish.

## 5. Conclusions

Our findings suggest that the molecular changes that occur during early zebrafish exposure to DEHP may be responsible for the disrupted reproduction seen in adult female zebrafish. We show that exposure to DEHP impairs reproduction by disturbing PGC migration and the establishment of reproductive neuroendocrine regulation. By targeting *cxcr4b* and *cxcr7b* expression, DEHP impairs the distribution of PGCs. DEHP also impairs PGC-specific *dazl* expression, with possible consequences for fertility. DEHP induces the expression of *cyp19a1a* and *lhb* and inhibits the expression of *esr1* and *esr2a*, therefore negatively affecting the developing neuroendocrine regulation of reproduction. The estrogenic potency of DEHP was further demonstrated by molecular docking analysis, which indicated that the main DEHP metabolite, MEHP, can bind to all three zebrafish nuclear estrogen receptors.

## Figures and Tables

**Table 1:** A list of primer sequences, primer efficiencies, amplicon length, and accession numbers.

Gene Name	Forward and Reverse Primer Sequence (5′ ⟶ 3′)	Efficiency (%)	Amplicon Length (bp)	Accession Number
*elf1a*	F CCTGGGATGAAACAGCTGATC R GCTGACTTCCTTGGTGATTTCC	96.5	100	NM_131263.1
*cxcr4b*	F CTTACTTACCCGCAGGAGGC R CCAGGCAGCAAAAAGCCAAT	91	83	NM_131834.1
*cxcr7b*	F TTCTCCAGGCATCGGAATCT R TCACACTCATCTTGGTCCGT	101	113	NM_001083832.1
*sdf-1a*	F ATTCGCGAGCTCAAGTTCCT R ATATCTGTGACGGTGGGCTG	99	214	NM_178307.2
*esr1*	F GTCTGGTCGTGTGAGGGATG R CCCTCCGCGATCTTTACGAA	101	189	NM_152959.1
*esr2a*	F ATACCGCCTTGCTCACTGTC R TCGGGATACTCGGACATGGT	103.5	141	NM_180966.2
*esr2b*	F TCATGTGAAGGGTGCAAGGC R TCCCTCCTCACACCACACTT	100.5	173	NM_174862.3
*ar*	F CACGAGCAGTGGTACCCG R TAGGCAGGTCCTTTGTGGAG	95	180	NM_001083123.1
*cyp19a1*	F CAGGGCATCATATTCAACTCAA R AGGTGGTGCAGATCTCCATAGT	95	112	NM_131154.3
*gnrh3*	F CGGTGGAAAAAGAAGCGTTGG R AACCTTCAGCATCCACCTCATT	99	143	NM_182887.3
*lhb*	F ATCGGTGGAAAAAGAGGGCTG R CTGGTGGACGGTGGAAAACG	104	115	NM_205622.2
*fshb*	F TGAGCGCAGAATCAGAATG R AGGCTGTGGTGTCGATTGT	105	105	NM_205624.2
*piwil1*	F TACCGCTGCTGGAAAAAGGT R CCTGCAATGACCCCTTCAGT	94	134	NM_183338.2
*piwil2*	F GGTGTCCATGTTCAGGGGTC R TGCTCCAATGATGGTGGGTT	94	130	NM_001365624.1
*nanos1*	F GCAATAAGCAGGAGCCCAAG R GGCTGGTCTTTGGAGAGAGG	96	199	NM_001305661.1
*dazl*	F TGCCAAGTATGGCTCAGTGAAA R ATTGCAGGTCCCAGTTTGAGT	106	162	NM_131524.1

**Table 2:** Estimated binding energies of best ranked models of estradiol and MEHP docked to Esr1, Esr2a, and Esr2b.

Receptor	Estimated Binding Energy for Estradiol [kCal/mol]	Estimated Binding Energy for MEHP [kCal/mol]
Esr1	−7.507	−5.477
Esr2a	−7.311	−6.019
Esr2b	−6.314	−5.45

## Data Availability

The original contributions presented in this study are included in the article. Further inquiries can be directed to the corresponding author.
